# Small, Cationic Antifungal Proteins from Filamentous Fungi Inhibit *Candida albicans* Growth in 3D Skin Infection Models

**DOI:** 10.1128/spectrum.00299-22

**Published:** 2022-05-02

**Authors:** Jeanett Holzknecht, Sandrine Dubrac, Sarah Hedtrich, László Galgóczy, Florentine Marx

**Affiliations:** a Biocenter, Institute of Molecular Biology, Medical University of Innsbruck, Innsbruck, Austria; b Department of Dermatology, Venereology and Allergology, Medical University of Innsbruck, Innsbruck, Austria; c Faculty of Pharmaceutical Sciences, University of British Columbia, Vancouver, British Columbia, Canada; d Department of Biotechnology, Faculty of Science and Informatics, University of Szeged, Szeged, Hungary; e Institute of Biochemistry, Biological Research Centre, Eötvös Loránd Research Network, Szeged, Hungary; Universidade de Sao Paulo

**Keywords:** 3D skin infection model, *Candida albicans*, full-thickness model, *Neosartorya* (*Aspergillus*) *fischeri*, *Penicillium chrysogenum*, antifungal proteins

## Abstract

The emerging resistance of human-pathogenic fungi to antifungal drugs urges the development of alternative therapeutic strategies. The small, cationic antifungal proteins (AFPs) from filamentous ascomycetes represent promising candidates for next-generation antifungals. These bio-molecules need to be tested for tolerance in the host and efficacy against fungal pathogens before they can be safely applied in humans. Testing of the efficacy and possible adverse effects of new drug candidates in three-dimensional (3D) human-cell based models represents an advantageous alternative to animal experiments. In, this study, as a proof-of-principle, we demonstrate the usefulness of 3D skin infection models for screening new antifungal drug candidates for topical application. We established a cutaneous infection with the opportunistic human-pathogenic yeast Candida albicans in a commercially available 3D full-thickness (FT) skin model to test the curative potential of distinct AFPs from Penicillium chrysogenum (PAF^opt^, PAFB, and PAFC) and *Neosartorya* (Aspergillus) *fischeri* (NFAP2) *in vitro*. All tested AFPs were comparably well tolerated by the skin models. The infected 3D models exhibited reduced epidermal permeability barriers, allowing C. albicans to colonize the epidermal and dermal layers, and showed increased secretion of the pro-inflammatory cytokine IL-6 and the chemokine IL-8. AFP treatment diminished the fungal burden and penetration depth of C. albicans in the infected models. The epidermal permeability barrier was restored and the secretion of IL-8 was decreased following AFP treatment. In summary, our study proves that the tested AFPs exhibit antifungal potential against cutaneous C. albicans infection in a 3D FT skin model.

**IMPORTANCE** Candida albicans represents one of the most prevalent opportunistic fungal pathogens, causing superficial skin and mucosal infections in humans with certain predisposing health conditions and life-threatening systemic infections in immunosuppressed patients. The emerging drug resistance of this human-pathogenic yeast and the limited number of antifungal drugs for prevention and treatment of infections urgently demands the identification of new antifungal compounds with novel mechanisms of action. Small, cationic antifungal proteins (AFPs) from filamentous fungi represent promising candidates for next-generation antifungals for topical application. These bio-molecules need to be tested for tolerance by the host and efficacy in pathogen clearance prior to being involved in clinical trials. In a proof-of-principle study, we provide evidence for the suitability of 3D human-cell based models as advantageous alternatives to animal experiments. We document the tolerance of specific AFPs and their curative efficacy against cutaneous C. albicans infection in a 3D skin model.

## INTRODUCTION

Fungal diseases affect ~1.35 billion people worldwide and kill ~1.6 million each year (1–3). Among fungi, *Candida* spp. are the most common commensals which asymptomatically colonize the human skin and mucosal surfaces (4). Stress factors and predisposing health conditions, e.g., medications, nosocomial infections, surgical interventions, parenteral nutrition, endocrine disorders, or a weak immune system disturb the human microbial ecosystem and allow *Candida* overgrowth which may result in non-fatal, but recurrent and often difficult-to-treat superficial infections that can impact the patient’s life (4). Immunocompromised patients, however, are at high risk for systemic candidiasis with high mortality rates when *Candida* invades deeper tissues and disseminates through the body (5, 6). Candida albicans represents one of the most prevalent opportunistic fungal pathogens in humans and is responsible for 80 to 90% of fungal infections in dermatology (7, 8). Body sites of high local humidity, occlusion, or maceration, such as intertriginal, submammary, and genitoanal skin, are particularly susceptible to cutaneous C. albicans infection (7, 9). Furthermore, patients undergoing broad-spectrum antibiotic treatment or suffering from immune dysregulation and specific skin inflammatory diseases, such as atopic dermatitis, psoriasis, or chronic mucocutaneous candidiasis, experience increased skin colonization with C. albicans, which exacerbates the inflammatory condition in the skin (10). In contrast to the numerous available antibacterial therapeutics, antifungal drugs are limited in number and often have strong, unpleasant side effects while showing insufficient therapeutic success. This is because fungi are eukaryotes and only few drug targets exist which are unique to fungi. Commonly, cutaneous candidiasis responds well to topical treatment with azoles, echinocandins, and the polyene drug nystatin, with mycological cure rates of 70 to 100% (11, 12); however, topical therapy may fail in immunocompromised patients and systemic treatment may be needed, whereupon the less-tolerated polyene amphotericin B is administered in salvage therapy (9, 13). The increase in the number of azole-resistant C. albicans strains further complicates treatment and compromises therapeutic success (14, 15). These facts drive the urgency of searching for alternative antifungal therapeutic agents (16, 17).

One promising source for novel antifungal compounds are filamentous fungi, which secrete small, cationic antifungal proteins (AFPs) (18, 19). In particular, members of the class Eurotiomycetes of the phylum Ascomycetes have been put in the spotlight, with several studies addressing their potential in future antifungal therapy development (19); for example, the three Penicillium chrysogenum antifungal proteins PAF, PAFB, and PAFC (20–22), and *Neosartorya* (Aspergillus) *fischeri* antifungal protein 2 (NFAP2 [23]). These specific AFPs stand out for their anti-*Candida* activity (22, 24–27). Whereas NFAP2 kills C. albicans rapidly, presumably by plasma membrane permeabilization (23), the P. chrysogenum AFPs are not canonical membrane active proteins. PAF, PAFB, and PAFC are first taken up by the fungal cell, then unfold their toxicity in the cytoplasm by inducing reactive oxygen species and permeabilizing yeast cell membranes (20, 22, 25). In the case of PAF, we observed the perturbation of the cytoplasmic calcium concentration and the induction of regulated cell death in fungi (28, 29).

Notably, AFPs possess the highly conserved structural motif “γ-core,” which has been shown to influence antifungal activity. For example, amino acid substitutions in the γ-core of PAF created the variant PAF^opt^, which has an elevated overall net charge and improved anti-yeast activity against C. albicans (20). The possibility of improving antifungal activity by rational design makes these AFPs interesting next-generation antifungals. The cation sensitivity of AFPs, however, limits their systemic administration; nevertheless, they may be candidates for the development of drugs for topical application to treat cutaneous *Candida* infection (19, 22, 25).

For the development of alternative antifungal strategies, AFPs should be extensively tested and characterized before therapeutic application. State of the art is the translation from *in vitro* cell culture to animal models. The discussion on reducing animal testing has been continuing for years, first defined by Russel and Burch in 1959 as the 3Rs: reduction, refinement, and replacement of animal models (30). Since then, major efforts have been put into advancing biomimetic and physico-chemically accurate tissue equivalents. One important step forward in this respect has been made by introducing three-dimensional (3D) full thickness (FT) skin models (31–35). These bilayered tissue constructs are cultured with primary human epidermal keratinocytes (hEK) and dermal fibroblasts (hDF), which differentiate into the distinct layers of human skin when cultured in an air-liquid interphase (ALI). These models can be purchased commercially and represent a feasible alternative to animal testing for pathophysiological studies and pharmacological investigations (36, 37). Some publications reported on the use of human 3D FT models to study the fungal infection of the skin and test new antifungal compounds (38–40).

In this study, we used the commercially available Phenion FT human skin model, which comprises an epidermis and a dermis and exhibits tissue architecture, physiological properties, and metabolic competence similar to that of normal human skin (33). This model was infected with C. albicans and tested for the histology, epidermal permeability barrier, and secretion of inflammatory cytokines in response to the fungal challenge. For anti-*Candida* compound testing, we selected four ascomycetous AFPs, PAF^opt^, PAFB, PAFC, and NFAP2, which have been previously reported to effectively inhibit the growth of C. albicans *in vitro* at μM concentrations (19–24). Here, we could prove the suitability of 3D FT human skin models for the testing of candidate AFPs against cutaneous candidiasis. This represents one step towards reducing the number of animal models and paving the way for future drug development.

## RESULTS

### Establishment of *C. albicans* infection in a 3D FT skin model.

**FIG 1:**
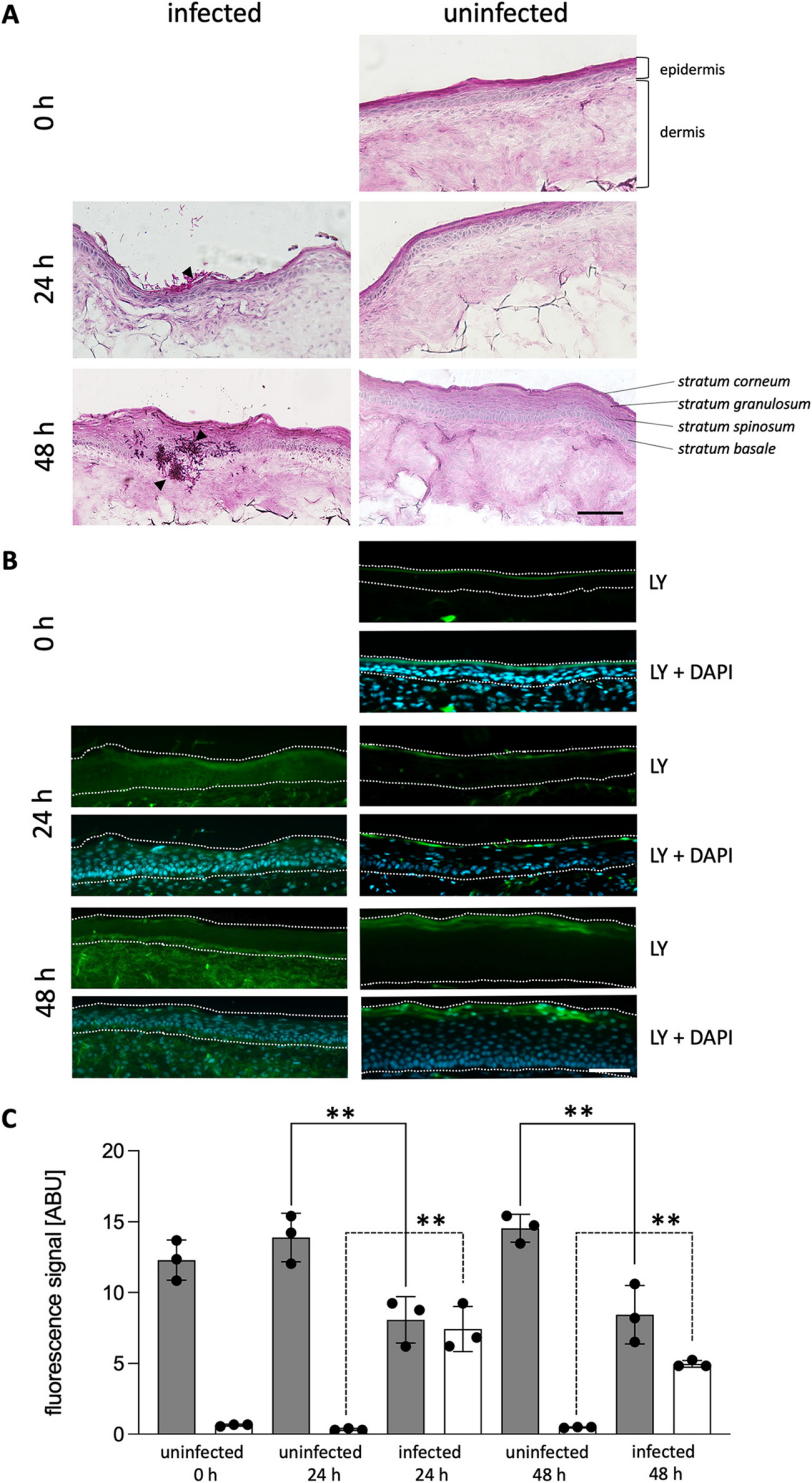
C. albicans infection of three-dimensional (3D) full-thickness (FT) skin models. Skin models infected with C. albicans (infected) and uninfected models (uninfected) were analyzed over 48 h of cultivation at 32°C, 5% CO_2_. (A) Cryo-sections were stained with periodic acid-Schiff (PAS) before microscopy. Arrowheads indicate C. albicans cells. (B) Here, 200 μL of Lucifer yellow (LY, 1 mg · mL^−1^) was topically applied for 2 h at 32°C, 5% CO_2_ before the models were harvested. Cryo-sections were mounted with Fluoroshield with 4′,6-diamidino-2-phenylindole (DAPI) for analysis of the epidermal permeability barrier. Dotted lines delineate the epidermis for LY and DAPI. (C) The LY fluorescence signal intensity was quantified in the *stratum corneum* (gray bars) and the viable epidermis (white bars) of the uninfected and infected models, and is depicted in arbitrary units (ABU). Bars represent the mean ± standard deviation (SD; *n* = 3). Asterisks indicate significant differences of the sample means of ABU in the *stratum corneum* and the viable epidermis of uninfected versus infected models after 24 h and 48 h of incubation (one-way analysis of variance [ANOVA] with Tukey’s honestly significant difference [HSD] test; **P < 0.005). Scale bars, 100 μm.

In this study, we utilized 3D FT skin models, which mimic the *in vivo* composition of the human skin. They displayed well-developed epidermal and dermal layers (Fig. 1A; Fig. S1A in the supplemental material) and the presence of specific tight-junction markers in the epidermal layers, i.e., the transmembrane proteins claudin-1 and occludin and the cytoplasmic plaque protein ZO-1 evidenced the formation of a functional paracellular barrier (Fig. S2) (41, 42). To establish the infection model, special care was taken to topically apply 200 viable colony forming units (CFU) of C. albicans CBS 5982 and evenly distribute the complete inoculum over the epidermal surface (1.4 cm^2^) of each model using a glass applicator (Table S1 in the supplemental material). The development of infection was monitored over a course of 48 h in models cultivated at 32°C in 5% CO_2_; uninfected models served as controls. The fungal burden was histologically documented with periodic acid-Schiff (PAS) and Grocott-Gömöri’s methenamine silver (GMS) staining, as shown in Fig. 1A and Fig. S1B, respectively. At 24 h postinfection, C. albicans cells had predominantly colonized the *stratum corneum*, whereby some hyphae had invaded the *stratum granulosum* (Fig. 1A, PAS; Fig. S1B, GMS). Further incubation for another 24 h revealed deeper hyphal penetration, reaching as far as the dermal layer, and massive colonization of the tissue (Fig. 1A; Fig. S1B).

The infection of the model was further proven by analysis of the *stratum corneum* permeability barrier using the fluorescent dye Lucifer yellow (LY). The uninfected models retained LY in the *stratum corneum*, which demonstrated no adverse effects of moisturizing the epidermal surface with phosphate-buffered saline (PBS) for 48 h (Fig. 1B). The colonization of the models with C. albicans caused LY to penetrate the *stratum corneum* and reach the dermis, indicating that infection with the pathogen dampened the outside-in permeability barrier (Fig. 1B, LY). This observation was further verified by a substantial increase in LY fluorescence signal intensity in the viable epidermis and a decrease in signal intensity in the *stratum corneum* of the infected skin models compared to that in the uninfected models (Fig. 1C). No adverse effects on tissue integrity could be otherwise detected, as documented with PAS (Fig. 1A) and hematoxylin and eosin (H&E) staining (Fig. S1). To prove that the infection had established only from the tops of the tissue models, and not from possible contamination of the culture medium, aliquots of conditioned culture medium (CCM) collected from the model culture wells were spread on Sabouraud agar (SBA) and Lysogeny broth (LB) agar plates. Neither fungal nor bacterial growth was observed after incubation of the respective plates (Fig. S3).

**FIG 2:**
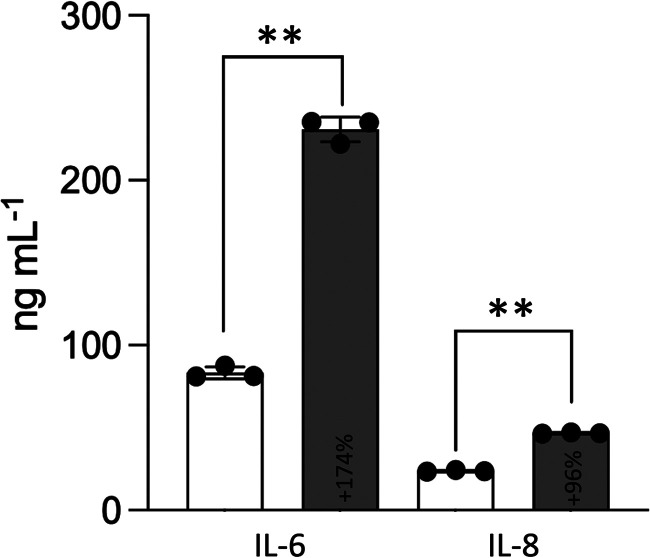
Release of IL-6 and IL-8 into the conditioned culture medium (CCM) of 3D FT skin models infected with C. albicans. Levels of cytokine secreted into the CCM of skin models cultivated for 48 h without (white bars) and with C. albicans infection (gray bars) were quantified by enyzme-linked immunosorbent assay (ELISA). Bars represent the mean ± SD (*n* = 3). Asterisks indicate the significant differences of the sample means of cytokine secretion between infected and uninfected models (one-way ANOVA with Tukey’s HSD test; **P < 0.005). The % change in cytokine concentration in response to C. albicans infection compared to that in the uninfected control (100%) is indicated in the respective bars.

To follow the induction of the innate immune response in the skin model, the secreted (pro-) inflammatory cytokines IL-6 and IL-8 were quantified in CCM collected after 48 h of incubation. IL-6 and IL-8 are expressed in keratinocytes and fibroblasts in response to fungal infection and secreted in sufficient amounts to be detectable by enzyme-linked immunosorbent assay (ELISA) in the CCM of skin models (38, 40, 43). As shown in Fig. 2, the secretion of IL-6 and IL-8 was highly elevated upon C. albicans infection compared to that in the uninfected control, indicating the induction of an inflammatory response in the models.

### Toxicity testing of AFPs in 3D FT skin models.

Before testing the impact of AFPs in the 3D FT skin models, we verified the *in vitro* inhibitory concentration reducing fungal growth by ≥90% (IC_90_) values on the test strain C. albicans CBS 5982 in broth microdilution assays, applying the antifungal compounds in 2-fold serial dilutions. As listed in Table S2 in the supplemental material, the IC_90_ values were 8.2 μg · mL^−1^ (1.3 μM) for PAF^opt^, 6.5 μg · mL^−1^ (1 μM) for PAFB, 16.6 μg · mL^−1^ (2.5 μM) for PAFC, and 2.2 μg · mL^−1^ (0.4 μM) for NFAP2. The IC_90_ of fluconazole (FLC) was 2.0 μg · mL^−1^ (6.4 μM) when it was tested under the applied experimental conditions.

**FIG 3:**
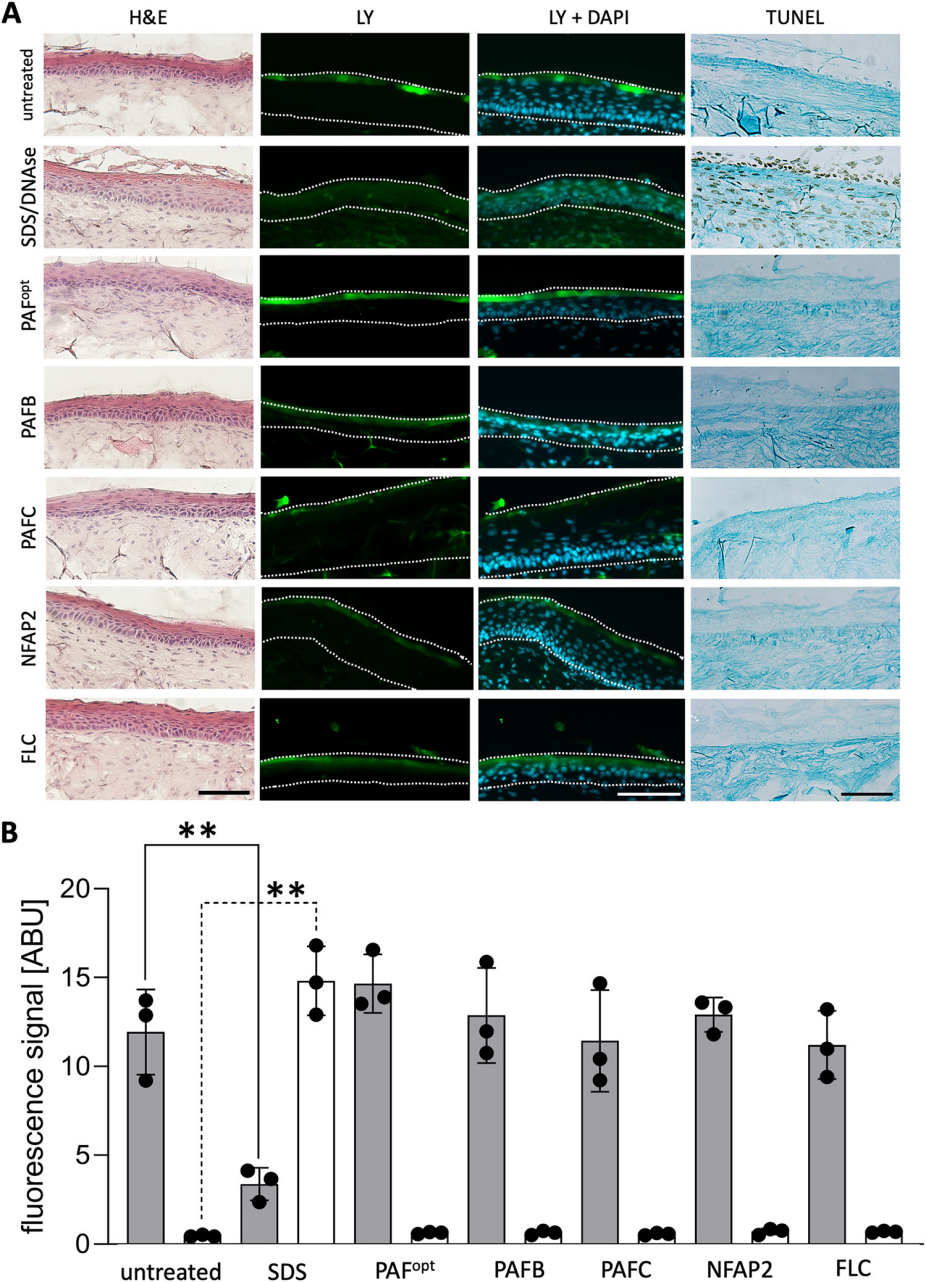
Evaluation of the impact of antifungal proteins (AFPs) on the permeability barrier of 3D FT skin models. (A) AFPs (PAF^opt^, PAFB, PAFC [18 mg · mL^−1^ each]; NFAP2 [6.4 mg · mL^−1^]) were applied in 25-μL aliquots onto 3D FT skin models, respectively. Twenty-five μL sterile ddH_2_O was applied to the untreated control and 25 μL fluconazole (FLC, 0.02 mg · mL^−1^) was used for comparison. After incubation for 24 h at 32°C, 5% CO_2_, 200 μL LY (1 mg · mL^−1^) was topically applied for 2 h at 32°C, 5% CO_2_. A model exposed to 1% SDS (wt/vol) for 60 min served as a toxicity control. Models were then washed with phosphate-buffered saline (PBS) and harvested, and cryo-sections were stained with either hematoxylin and eosin (H&E) or TdT-mediated dUTP-biotin nick end labeling (TUNEL) before microscopy. For TUNEL, a DNase-treated control was included. Dotted lines delineate the epidermis for LY and DAPI. (B) The LY fluorescence signal intensity was quantified in the *stratum corneum* (gray bars) and viable epidermis (white bars) of the infected models after antifungal treatment, and is depicted in arbitrary units. Uninfected models without treatment served as controls. Bars represent the mean ± SD (*n* = 3). Asterisks indicate significant differences of the sample means of ABU of the uninfected models treated with PAF^opt^, PAFB, PAFC, NFAP2, or FLC compared to those of the uninfected, untreated control (uninfected) (one-way ANOVA with Tukey’s HSD test; **P < 0.005). Scale bars, 100 μm.

In previous studies, we observed that *Candida* spp. growing in a biofilm are less susceptible to AFPs than planktonic cells *in vitro* (22, 26, 27). Therefore, we applied the highest possible concentrations of AFPs to prove their tolerance in the skin models and their antifungal efficacy in the infected 3D FT skin models. To this end, we analyzed histology and cytokine secretion profiles. We included the anti-*Candida* azole drug FLC, which is water-soluble at low concentrations, to compare its effect with that of the AFPs when applied in an aqueous solution (38). As a control for tissue damage, we used the detergent sodium dodecyl sulfate (SDS). H&E staining revealed no changes in the morphology or thickness of the distinct skin layers of the models in response to AFP and FLC treatment, respectively (Fig. 3A). Instead, the application of SDS resulted in a peeling of the upper epidermal layers (Fig. 3A). LY accumulated in the *stratum corneum* of the AFP- and FLC-exposed models, similar to the untreated control which was exposed to double-distilled water (ddH_2_O, Fig. 3A), showing no alterations to an intact outside-in permeability barrier. In contrast, the SDS treatment resulted in increased permeability, causing the dye to penetrate into the epidermal layers of the skin model (Fig. 3A). The significant decrease in LY fluorescence signal intensity in the *stratum corneum* and increase in the viable epidermis of the skin models treated with SDS nicely reflected the histochemistry data (Fig. 3B).

To study the impact of the treatments on the single-cell level, we applied the TdT-mediated dUTP-biotin nick end labeling (TUNEL) method to detect apoptotic nuclei by labeling DNA strand breaks with a deoxynucleotidyl transferase-biotin-streptavidin-horseradish peroxidase-conjugate. To visualize the skin layers, the TUNEL-stained samples were counterstained with Light Green. A negative (untreated) and positive (DNase treatment) control were included. As depicted in Fig. 3A (TUNEL), tissue sections treated with DNase showed brown nuclei, which are characteristic of apoptotic cells. In contrast, treatment with AFPs and FLC did not induce any programmed cell death in the tissue, confirming the tolerance of the 3D FT skin models for the AFPs and FLC.

Next, we tracked the penetration of AFPs in the 3D FT skin models by applying Bd-labeled AFPs onto the models. All AFPs were detected only in the *stratum corneum* (Fig. S4).

**FIG 4:**
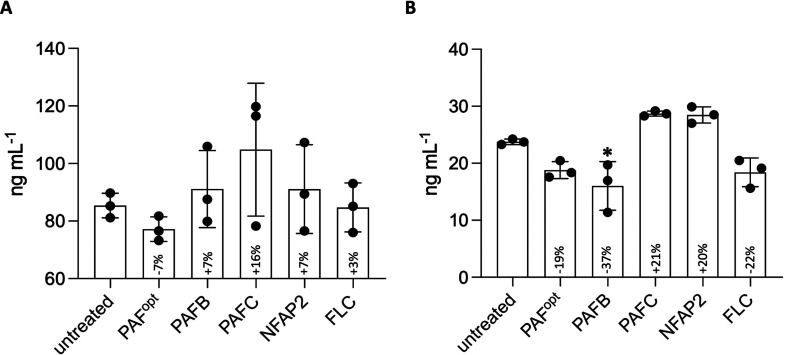
Release of IL-6 and IL-8 into the CCM of 3D FT skin models after antifungal treatment. The release of (A) IL-6 and (B) IL-8 into the CCM of 48-h-old uninfected skin models that were topically treated with 25 μL AFPs (PAF^opt^, PAFB, PAFC [18 mg · mL^−1^ each]; NFAP2 [6.4 mg · mL^−1^]) or FLC (0.02 mg · mL^−1^) for 24 h was quantified by ELISA. ddH_2_O (25 μL) was applied to the uninfected, untreated control (untreated). Bars represent the mean ± SD (*n* = 3). Asterisks indicate significant differences of the sample means of models after antifungal treatment compared to those of the untreated control (one-way ANOVA with Tukey’s HSD test; *P < 0.05). The % change in cytokine concentration in response to antifungal treatment compared to the cytokine concentration in the untreated control (100%) is indicated in the respective bars.

Cytokine quantification in the CCM by ELISA revealed a mild modulation of IL-6 and IL-8 secretion, dependent on the antifungal compound applied. As shown in Fig. 4, PAFC induced the strongest secretion of IL-6 (+16%), whereas PAFB resulted in the highest decrease of IL-8 secretion (–37%).

### Efficacy of AFPs in impeding *C. albicans* infection in an 3D FT skin model.

**FIG 5:**
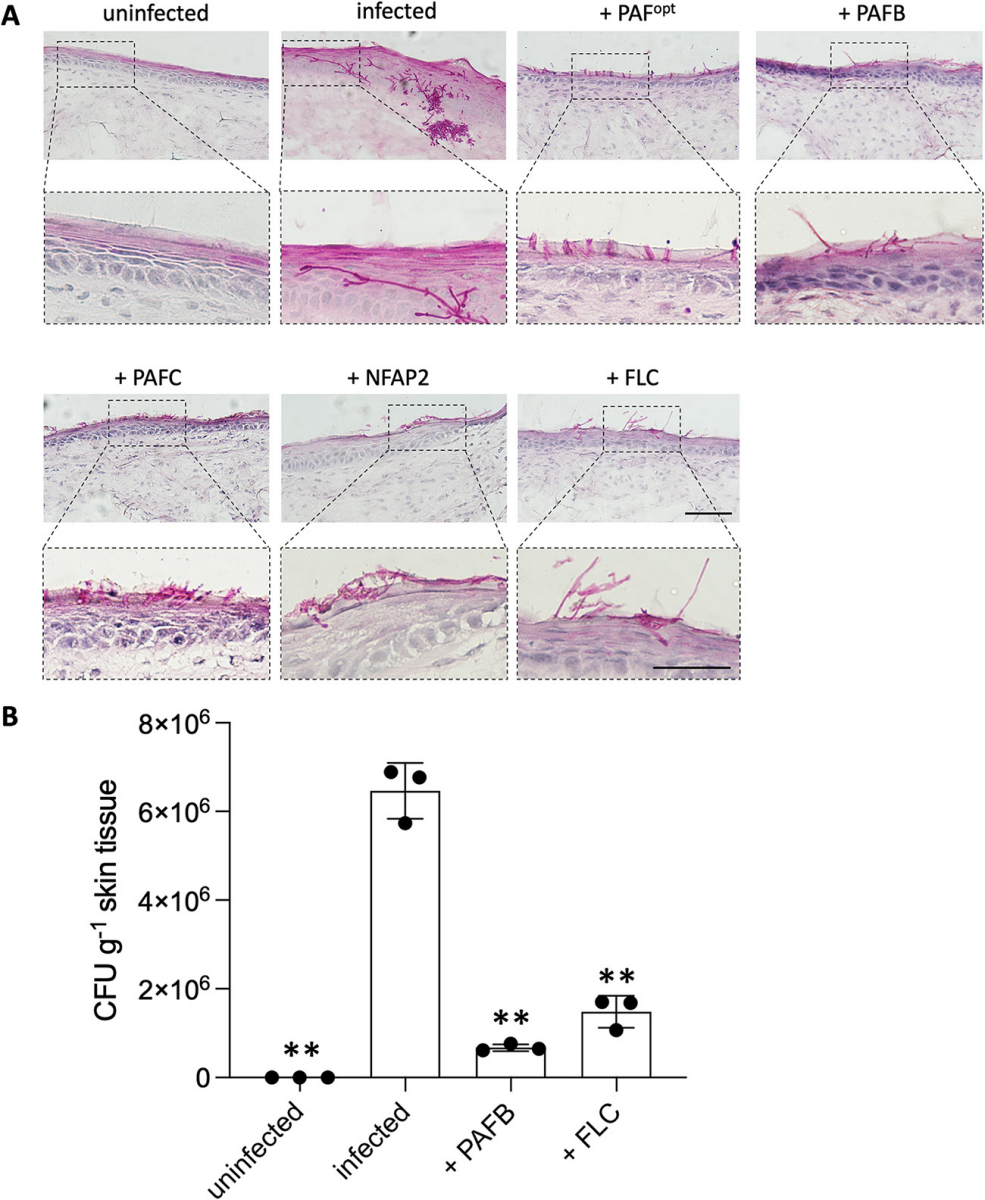
Analysis of the antifungal efficacy of AFPs in C. albicans-infected 3D FT skin models. Skin models infected with C. albicans were cultured for 24 h at 32°C, 5% CO_2_ before 25 μL AFPs (PAF^opt^, PAFB, PAFC [18 mg · mL^−1^ each]; NFAP2 [6.4 mg · mL^−1^]) were topically applied and incubated for a further 24 h under the same cultivation conditions. As a control, 25 μL ddH_2_O was applied to uninfected and C. albicans-infected models, respectively. The same volume of FLC (0.02 mg · mL^−1^) was applied for comparison. (A) Fungal burden of the skin models was visualized by PAS staining. Magnification of the insets are shown below the respective microscopic images. (B) C. albicans burden in the infected skin models after treatment with PAFB or FLC was quantified and depicted as colony forming units (CFU) per skin tissue weight (g). Uninfected and infected models without treatment served as controls. Bars represent the mean ± SD (*n* = 3). Asterisks show significant differences of the sample means of infected and antifungal-treated (PAFB, FLC) or uninfected, untreated (uninfected) models compared to those of the untreated infection control (infected) (one-way ANOVA with Tukey’s HSD test; **P < 0.005). Scale bars, 100 μm.

**FIG 6:**
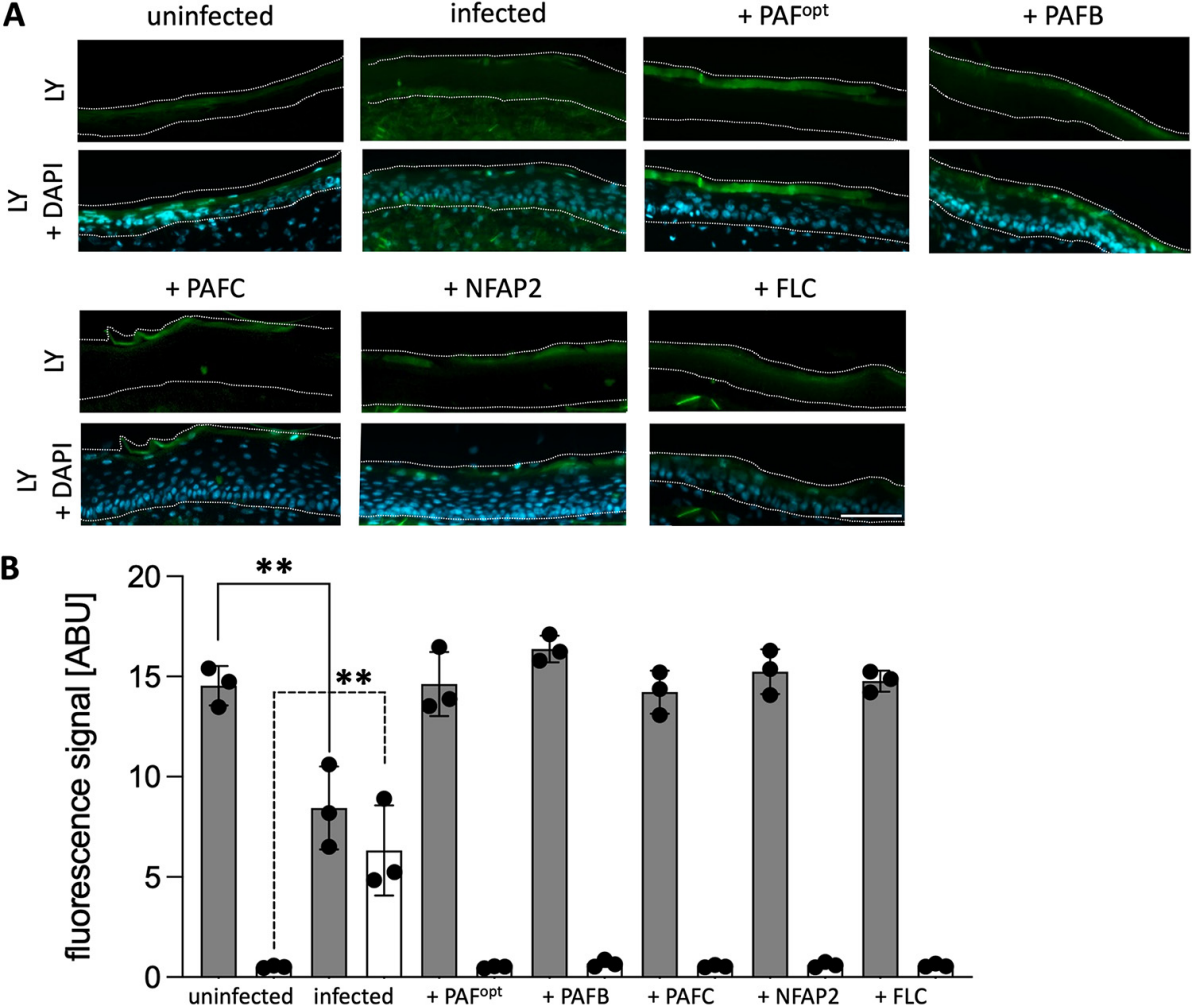
Analysis of the effect of AFP treatment on the permeability barrier of C. albicans-infected 3D FT skin models. Skin models infected with C. albicans were cultured for 24 h at 32°C, 5% CO_2_ before 25 μL AFPs (PAF^opt^, PAFB, PAFC [18 mg · mL^−1^ each]; NFAP2 [6.4 mg · mL^−1^]) were topically applied and incubated for a further 24 h under the same cultivation conditions. As a control, 25 μL ddH_2_O was applied on uninfected/untreated (uninfected) and infected/untreated (C. albicans) models, respectively. The same volume of FLC (0.02 mg · mL^−1^) was applied for comparison. (A) A 200-μL volume of LY (1 mg · mL^−1^) was topically applied on the models for 2 h at 32°C, 5% CO_2_ before harvesting. Cryo-sections were mounted with Fluoroshield with DAPI for analysis of the epidermal permeability barrier. Dotted lines delineate the epidermis for LY and DAPI. (B) LY fluorescence signal intensity was quantified in the *stratum corneum* (gray bars) and the viable epidermis (white bars) of the infected models after antifungal treatment and is depicted in ABU. Uninfected and C. albicans infected models without treatment served as controls. Bars represent the mean ± SD (*n* = 3). Asterisks indicate significant differences of the sample means of infected, antifungal-treated (PAF^opt^, PAFB, PAFC, NFAP2, FLC) or infected, untreated (infected) models compared to those of the uninfected, untreated control (uninfected) (one-way ANOVA with Tukey’s HSD test; **P < 0.005). Scale bars, 100 μm.

We treated C. albicans-infected 3D FT skin models with AFPs to determine their efficacy at reducing fungal burden compared to that in the infected controls with no AFP treatment. To establish the infection, the 3D FT skin models were first inoculated with 200 CFU of C. albicans and cultivated for 24 h in 5% CO_2_ at 32°C before the AFPs were applied. After an additional incubation of 24 h with AFPs, the models were analyzed. An uninfected, untreated model and an infected model without antifungal treatment were used as controls. The H&E staining revealed no differences in overall tissue structure between all treatment regimens (Fig. S5). However, PAS- (Fig. 5A) and GMS staining (Fig. S5) of the infected, untreated models confirmed the colonization of models with C. albicans. A morphological transition between the yeast and hyphal cell forms and fungal invasion of the epidermal and dermal layers were observed (Fig. 5A). In contrast, the infected models treated with AFPs or FLC exhibited a significantly reduced fungal burden (Fig. 5A; Fig. S5), and the few remaining C. albicans cells pertained in the *stratum corneum* after AFP or FLC treatments without penetrating deeper into the skin model (Fig. 5A; Fig. S5). The tested AFPs and FLC appeared similarly effective in inhibiting C. albicans growth in the 3D FT skin models. We therefore selected PAFB as one exemplary AFP and FLC as a control to correlate these results with the quantification of the number of viable C. albicans cells in the models. We determined CFU counts in the infected models after treatment with PAFB or FLC compared to that in the infected but untreated models. Figure 5B shows that the CFU counts reflected the histochemistry results. The curative effect of AFPs was further evidenced by the restoration of the outside-in permeability barrier in the infected models after antifungal treatment (Fig. 6). While C. albicans infection resulted in the loss of barrier integrity, as shown by the detection of LY throughout the whole epidermis until the dermal layer, LY was mostly retained in the *stratum corneum* of the infected models treated with AFPs or FLC (Fig. 6A). This result corresponded well with the quantification of LY fluorescence signal intensity, which was the strongest in the *stratum corneum* of the infected skin models after antifungal treatment and in the uninfected control model (Fig. 6B). In contrast, the signal intensity was significantly decreased in the *stratum corneum* and increased in the viable epidermis of the infected, untreated models (Fig. 6B). Notably, no microbial growth could be detected in the CCM of the models, regardless of treatment regimens (Fig. S6).

**FIG 7:**
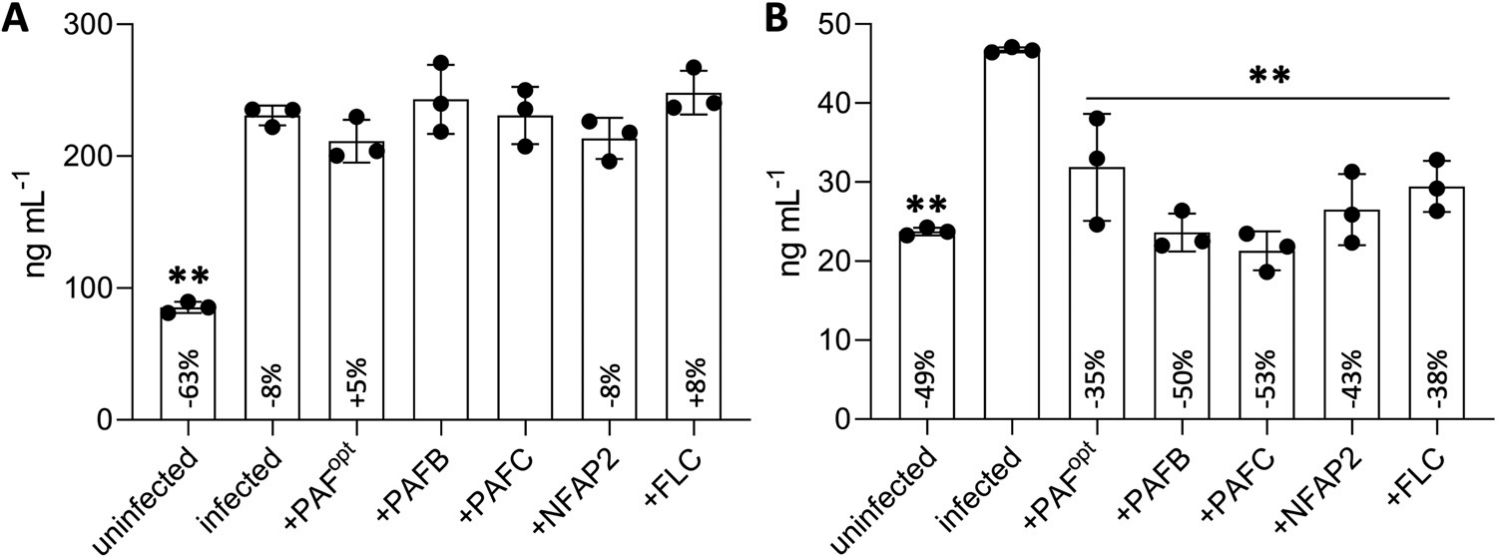
Release of IL-6 and IL-8 into the CCM of 3D FT skin models after infection with C. albicans and subsequent antifungal treatment. The release of (A) IL-6 and (B) IL-8 into the CCM of 48-h-old infected skin models which were topically treated with AFPs or FLC for 24 h was quantified by ELISA. ddH_2_O (25 μL) was applied to uninfected and infected skin models, which served as untreated controls. Bars represent the mean ± SD (*n* = 3). Asterisks indicate significant differences of the sample means of infected, antifungal-treated (PAF^opt^, PAFB, PAFC, NFAP2, FLC) or uninfected, untreated (uninfected) models compared to those of the untreated infection control (infected) (one-way ANOVA with Tukey’s HSD test; **P < 0.005). The % change in cytokine concentration in response to antifungal treatment is indicated relative to the cytokine concentration in the infected, untreated control, which was set at 100%. For comparison, the cytokine concentration and % change in the uninfected, untreated models is shown relative to that of the untreated infection control.

Next, we analyzed the release of IL-6 and IL-8 into the CCM of the infected models exposed to AFPs or FLC to assess the outcome of a 24-h antifungal treatment. The CCM of uninfected/untreated and infected/untreated models served as controls (Fig. 7). A significant reduction in IL-8 was detected after the application of AFPs or FLC, whereas antifungal treatment in the infected models resulted in no significant decrease in IL-6 secretion (Fig. 7).

## DISCUSSION

The human skin is a physicochemical barrier which protects the body from pathogenic microorganisms while being a habitat to a vast variety of microorganisms which are part of the commensal microbiota. The yeast C. albicans is a harmless commensal on healthy skin and epithelial surfaces, but can become a pathogen under specific predisposing conditions (44–46). The fine line that separates the commensal from the pathogenic state of C. albicans requires tight regulation of the host’s immune response on the one hand and C. albicans evasion strategies on the other (47, 48). Weakening of the skin immune system and/or inefficient antifungal therapy may lead to the overgrowth of this opportunistic fungal pathogen, deeper penetration of the dermal layers, dissemination of the pathogen, and potentially systemic infection, the latter being a life-threatening condition (49).

Currently, therapeutic options are narrow, and the main limitations for the development of new antifungal drugs are (i) the small number of drug targets which are unique to fungi and absent in the host, (ii) potential toxicity to the patient, (iii) the development of resistance mechanisms in fungal pathogens, (iv) the long duration and high cost of pre-clinical studies in animal models, and (v) the discrepancies in therapeutic responses between animal models and patients (50–52).

AFPs from filamentous fungi represent promising next-generation drug candidates (19) owing to their high antifungal activity against C. albicans and the tolerance of mammalian cells to such molecules (20, 22, 25, 26). The first reports on the *in vivo* efficacy of AFPs against fungal infections documented their applicability. In a murine pulmonary aspergillosis model, the survival of infected animals was prolonged when a combinatorial therapy with PAF and amphotericin B was administered (53). Moreover, NFAP2, in combination with FLC, successfully decreased the cell count of a FLC-resistant C. albicans clinical isolate in a murine model of vulvovaginal candidiasis (26). Both AFPs were well tolerated by the animal hosts (26, 54).

In the early phases of drug development, however, the use of large numbers of animals to screen potential candidate compounds for their toxicity, delivery, and efficacy is limited by ethical, regulatory, practical, or economic reasons. To test potential drugs which are intended for topical application to treat skin infections, researchers have relied on *ex vivo* animal models which predominantly employ pig and rodent skin. However, human and animal skin vary, e.g., in structure, barrier properties, and lipid composition in the *stratum corneum*, which results in high failure rates in clinical testing and poor transferability of animal data to humans (32, 52). Due to the limited availability of *ex vivo* human skin as the “gold-standard” experimental model, major effort is being put into developing alternatives which more closely mimic the human *in vivo* environment (55, 56).

3D FT skin models are a powerful and easy-to-handle alternative to animal models, allowing studies to be performed under standardized conditions with controlled parameters. They accelerate the process of screening for the efficacy and cytotoxicity of new antifungal drug candidates, thus helping to lower the number of animals sacrificed (57, 58).

While 3D reconstructed human epidermis (RHE) represents a simple model composed only of primary human hEK, which differentiate into the epidermal layers *stratum corneum*, *stratum granulosum*, *stratum spinosum*, and *stratum basale* when cultured in ALI, the 3D FT skin models present an additional dermal layer. Thus, 3D FT models may be superior to RHE. It is known that the dynamic cross-talk between keratinocytes and fibroblasts is crucial for the maintenance of skin homeostasis and regulates epidermal morphogenesis (59). Furthermore, other cell types, such as T-cells or dendritic cells, can be integrated into 3D FT skin models to provide more complete models for evaluating inflammatory mechanisms and immune responses involved in skin infection, disease, and treatment regimens (38, 60).

The two-component Phenion 3D FT skin model used in our study nicely recapitulates the main features of native human skin (Fig. 1), and the cells of distinct epidermal layers express proteins constituting tight junctions (Fig. S2), indicating an unaltered paracellular barrier (41, 42). Despite its relative simplicity (e.g., lack of immune cells), it represents a useful tool for non-clinical screening protocols, as reported in this study.

The 3D FT skin models exhibited signs of infection predominantly in the *stratum corneum* at 24 h postinfection with C. albicans. The epithelial damage was detectable by the loss of the outside-in permeability barrier, and fungal tissue colonization was accompanied by a transition from the yeast to the hyphal cell form. As reported earlier, this change in morphology occurs within the first hours following infection of epithelial cells (61). The ability to form hyphae is considered to be one main virulence factor of C. albicans, because this specialized cell form actively penetrates the host tissue (62–64). Prolonged incubation, i.e., 48 h, resulted in deeper penetration of C. albicans into the skin models, leading to massive colonization of the epidermal and dermal layers (Fig. 1).

As previously reported, colonization of the *stratum corneum* with yeast cells does not elicit an immune response, whereas invasion of the skin by yeast hyphae triggers the production of pro-inflammatory cytokines in living granular keratinocytes through the recognition of fungal molecular patterns by Toll-like receptors (65–68). In line with this, in our model of skin infection with C. albicans, an inflammatory, innate response was observed and characterized by the increased secretion of IL-6 and IL-8 into the CCM (Fig. 2). The chemokine IL-8 recruits phagocytic cells, e.g., neutrophils, macrophages, and dendritic cells, to the site of inflammation or infection (69, 70). IL-6 is a multifunctional cytokine involved in pro- and anti-inflammatory regenerative processes. It activates the adaptive immune system by recruiting mononuclear cells and preventing T-cell apoptosis, but it also supports cell proliferation, tissue regeneration in wound healing, and inhibition of epithelial cell apoptosis (71, 72). A two-component 3D skin model, however, lacks a microbiota and immune cells, and the activation of cytokine release by living keratinocytes in response to invading fungi is not sufficient for pathogen clearance (38, 73). A protective immune response which also activates dermal fibroblasts and prevents C. albicans invasion requires supplementation of the model with phagocytic cells and/or naive or activated CD4^+^ T-cells (38).

In the current experimental setup, the topically applied AFPs remained in the *stratum corneum*, where they accumulated without inducing any adverse effects on the tissue structure or the epidermal barrier. The *stratum corneum* is relatively impermeable to aqueous solutions and many chemicals (74). When small molecules or peptides are topically applied, less than 3% of the compound amount penetrates the *stratum corneum* and is absorbed through the skin (75). This might limit AFP applicability, as penetration and accumulation of antifungals in deeper layers support efficient pathogen clearance. Therefore, intensive research should focus on the development of new and improved formulations, such as nanogels and lipid formulations, to increase drug absorption through the skin and avoid allergic skin reactions (76–80). The topical application of AFPs or FLC only mildly altered IL-6 and IL-8 secretion in the uninfected 3D FT skin models (Fig. 4). However, model morphology and the epidermal permeability barrier were not affected (Fig. 3). Moreover, neither histochemistry nor TUNEL- or LY-based analyses revealed any signs of skin irritation or tissue damage (Fig. 3). Together, these data indicate that the AFPs were well tolerated by the skin models.

When 3D FT skin models were first infected with C. albicans and then topically treated with AFPs (curative experimental setting), the invasion of the skin with the fungus regressed in a similar manner to that induced by FLC (Fig. 5A), and the number of viable cells was massively reduced (Fig. 5B). The skin permeability barrier, as shown by the retention of LY in the *stratum corneum*, was restored (Fig. 6). Moreover, the concentration of secreted IL-8 was significantly decreased in the infected models after antifungal treatment, confirming the reduced fungal burden and underlining the antifungal potential of all tested AFPs (Fig. 7B). Nevertheless, IL-6 levels remained elevated in all infected samples after exposure to AFPs or FLC compared to that in the infected, but untreated skin models (Fig. 7A). It is likely that, after antifungal treatment, residual C. albicans cells, yeast cell debris, or secreted fungal material still stimulated IL-6 production in the models (81).

In summary, we established a cutaneous C. albicans infection in a commercially available 3D FT skin model. We proved the good tolerance of the human skin models to specific AFPs isolated from filamentous ascomycetes and documented their antifungal efficacy against C. albicans after topical application. The 3D FT skin infection model with C. albicans is a valuable tool to screen new antifungal compounds, such as AFPs, for candidates with the highest therapeutic efficacy in single uses, and it could be also employed to test AFPs in combination with licensed antifungal drugs. Future work on modulation of the primary structure of AFPs by rational design, and on specific formulations to increase their penetration and absorption through the skin, further promise a rise in the efficacy of AFPs against cutaneous C. albicans infection.

## MATERIALS AND METHODS

### Antifungal compounds used in this study.

PAF^opt^, PAFB, PAFC, and NFAP2 were recombinantly produced in a P. chrysogenum-based expression system and purified as described previously (20–22, 24, 82). The anti-*Candida* azole drug FLC was purchased from Santa Cruz Biotechnology (Dallas, TX, USA). The IC_90_ was verified in broth microdilution assays as described by Sonderegger et al. (82). In brief, C. albicans CBS 5982 cells (1 × 10^4^ cells · mL^−1^) were grown in 10-fold diluted potato dextrose broth (0.1× PDB; Sigma-Aldrich, St. Louis, MO, USA) containing increasing concentrations of AFPs (0 to 10 μM), respectively, in a final volume of 200 μL for 24 h at 30°C under static conditions. For comparison, FLC was included (concentration range: 0 to 25 μM) using the same *in vitro* testing conditions. Fungal growth was evaluated spectrophotometrically by measuring the optical density at an absorbance wavelength of 620 nm (OD_620_) in a multi-mode plate reader (FLUOstar Omega, BMG Labtech, Ortenberg, Germany). The OD_620_ value of untreated cells served as a growth control and was set at 100%.

### 3D FT skin models.

The 3D FT skin models were purchased from Phenion (Henkel AG, Düsseldorf, Germany). These models are 1.4-cm diameter in size and built from hKE and hDF, which were isolated from juvenile foreskin derived from the same human donor and expanded in a 2D culture *in vitro*. The hDF are embedded into a sponge of chemically cross-linked collagen that mimics the *in vivo* dermal extracellular matrix, and hKE are seeded on top. After 1 week of cultivation under submersed conditions, the models are lifted to ALI for differentiation into distinct skin layers (33). The skin models were shipped and used for experiments at ALI day 6 (ALI 6). After arrival, the insert models were immediately transferred in a standing position to a CytoOne 12-well plate (Starlab, Hamburg, Germany) containing 0.8 mL pre-warmed (32°C) Phenion Air-Liquid Interface Culture Medium without antibiotics (Henkel AG) per well, and shortly equilibrated for 1 h at 32°C, 5% CO_2_. After the distinct treatments, models were cut out from the inserts and histologically analyzed. For each experimental setting, three skin models were used (*n* = 3). The conditioned culture medium was routinely checked for microbial contamination. To this end, 50 μL CCM was plated in duplicates on LB agar and SBA (Table S3 in the supplemental material), respectively, and incubated at 37°C for up to 96 h. Plates were documented with a Nikon D7000 digital camera. The remaining CCM was stored for further analyses at −80°C.

### Infection of 3D FT skin models with *C. albicans*.

C. albicans CBS 5982 was streaked on potato dextrose agar (PDA; Table S3). A single colony was then picked and incubated in liquid yeast extract peptone dextrose (YPD) medium (Table S3) for 24 h at 37°C on a rotary shaker. Cells were pelleted at 50 × *g* for 10 min, washed three times with PBS (pH 7.4) (Table S3), and resuspended in PBS to a final cell concentration of 1.6 × 10^4^ mL^−1^. To test yeast cell viability, 25 μL of the C. albicans suspension was plated onto SBA and incubated for 24 h at 37°C to count the CFU. The skin models were infected by spreading 200 CFU in PBS in 25-μL aliquots on top of the ALI 6 models using sterilized round glass applicators. To control the efficient application of C. albicans cells onto the model, the used applicators were dragged over SBA and the plates were incubated for 24 h at 37°C to count the CFU of cells that were not transferred onto the models. The models were incubated for 24 (ALI 7) and 48 h (ALI 8) at 32°C, 5% CO_2_ before analyses. PBS (25 μL) without C. albicans cells was applied onto the uninfected control models.

### Application of AFPs on 3D FT skin models.

For the analysis of the impact of AFPs on the uninfected 3D FT skin models, the same application and incubation conditions were used as described below for the treatment of C. albicans-infected models with APFs. This allowed comparison of the AFP effect between these two conditions over the course of the study. Thus, equilibrated ALI 6 models were first topically treated with 25 μL of PBS and incubated for 24 h at 32°C, 5% CO_2_. Then, at ALI 7, 25 μL of PAF^opt^, PAFB, PAFC (18 mg · mL^−1^; 2.7 to 2.9 mM), or NFAP2 (6.4 mg · mL^−1^; 1.2 mM) solved in sterile ddH_2_O, respectively, was topically applied to the models. This corresponded to 300 μg per cm^2^ of PAF^opt^, PAFB, and PAFC, respectively, and 107 μg per cm^2^ of NFAP2. As a negative control, 25 μL of sterile ddH_2_O without AFPs was applied. A model exposed to 25 μL FLC (0.02 mg · mL^−1^ in sterile ddH_2_O; 65 μM) was included for comparison, corresponding to 333 ng per cm^2^ of FLC (adapted from Kühbacher et al. [38]). The models were incubated for another 24 h at 32°C, 5% CO_2_ before harvesting at ALI 8. For penetration studies, the AFPs were replaced by AFPs labeled with the green fluorophore BODIPY FL EDA (Bd; Invitrogen, Waltham, MA, USA) as previously described (83).

### Treatment of *C. albicans*-infected 3D FT skin models with AFPs.

After infection of the ALI 6 models with C. albicans and incubation for 24 h at 32°C, 5% CO_2_, AFPs were topically applied. Therefore, 25 μL of PAF^opt^, PAFB, PAFC (18 mg · mL^−1^), or NFAP2 (6.4 mg · mL^−1^) solved in sterile ddH_2_O, respectively, was applied on ALI 7 models and spread using a round glass applicator. ddH_2_O was used as a negative control and 25 μL of FLC (0.02 mg · mL^−1^) included for comparison. The models were then further incubated for 24 h at 32°C, 5% CO_2_ until analyses were performed at ALI 8.

### Quantification of *C. albicans* in 3D FT skin models.

The CFU were determined to quantify the C. albicans burden in the infected 3D FT skin models that were exposed to antifungal treatment. For a proof-of-principle approach, we selected PAFB and FLC for the treatment. The C. albicans-infected and treated skin models were harvested, weighted, and homogenized using a mortar and pestle. One mL PBS was added to the homogenate and 50-μL serial dilutions were plated in triplicates onto SBA. Plates were incubated at 37°C for 24 h. Uninfected and infected models without treatment were used as negative and positive controls, respectively. The CFU were counted, and the growth reduction achieved by the antifungal treatment was related to tissue weight and compared to the CFU · g^−1^ tissue of the infected, untreated models (positive growth control).

### Analysis of the *stratum corneum* permeability barrier with Lucifer yellow.

To study the outside-in permeability barrier, 200 μL LY CH dilithium salt (1 mg · mL^−1^ in PBS, Sigma-Aldrich) was topically applied onto the ALI 8 models for 2 h at 32°C, 5% CO_2_ (84). A model exposed to 1% (wt/vol) SDS (Sigma-Aldrich) for 60 min prior to LY application was included as a control for increased skin permeability (85). After incubation, the models were washed three times with PBS to remove excess dye before the preparation of cryo-sections as described below.

### Histochemical analysis of the 3D FT skin models.

For histochemical analysis, the skin models were submerged in 22 × 22 mm Peel-A-Way disposable plastic tissue embedding molds (Science Services, Munich, Germany) containing FSC 22 frozen section compound (Leica Biosystems, Wetzlar, Germany) and flash-frozen in liquid nitrogen. Models were stored at least one night at −80°C before further processing. Samples were then cut into 7-μm sections using a Cryostat CM1850 (Leica Biosystems, Wetzlar, Germany) at −20°C against the direction of application (from dermis to epidermis) and put onto glass slides for subsequent histological staining. At least 30 sections were prepared from each skin model and analyzed for each histochemical staining.

### Hematoxylin and eosin staining.

H&E staining was conducted as described in standard protocols (86). In short, after the samples were fixed with 4% (vol/vol) paraformaldehyde (PFA), they were washed with PBS, hydrated in ddH_2_O, and stained with acidic Mayer’s Hematoxylin (Carl Roth, Karlsruhe, Germany) for 5 min with agitation. Sections were then rinsed for 5 min under tap water for bluing, counterstained for 30 s with Eosin Y solution (0.5% [wt/vol] in H_2_O, Carl Roth), and dehydrated through ascending alcohols before clearing with ROTI-Histol (Carl Roth) and mounting with ROTI-Histokit (Carl Roth).

### Periodic acid-Schiff staining.

After fixation in 4% (vol/vol) PFA, the samples were stained as described in the PAS staining protocol (Bio-Optica, Milan, Italy), with minor modifications (87). Briefly, samples were washed in ddH_2_O, oxidized for 10 min with 1% (vol/vol) periodic acid solution (Bio-Optica), hydrated in ddH_2_O, and then stained for 20 min with the Schiff reagent Hotchkiss McManus (Bio-Optica). Samples were then hydrated in ddH_2_O and incubated for 4 min with 0.5% (wt/vol) potassium metabisulfite (Bio-Optica). After washing, they were counterstained with acidic Mayer’s Hematoxylin (Carl Roth, Karlsruhe, German) and blued for 5 min in tap water. Samples were then dehydrated through ascending alcohols, cleared with ROTI-Histol, and mounted with ROTI-Histokit (Carl Roth).

### Grocott-Gömöri’s methenamine silver staining.

The GMS staining was based on GMS staining kit protocol (BioVision, Milpitas, CA, USA) with minor modifications (88). Briefly, after fixation in 4% (vol/vol) PFA, the samples were rinsed in ddH_2_O, incubated for 10 min in chromic acid solution (BioVision), rinsed once in tap water and twice with ddH_2_O, and treated with sodium bisulfite solution (BioVision) for 1 min to remove excess chromic acid. Sections were washed twice with ddH_2_O before incubating at 60°C in GMS working solution, pre-warmed at 60°C (Table S3). The samples were checked repeatedly until C. albicans cells appeared black or darkish brown under the microscope. Slides were then rinsed 4 times with ddH_2_O and lightened with gold chloride solution (BioVision) for 30 s. After the sections were incubated for 2 min in sodium thiosulfate solution (BioVision), they were rinsed in tap water and washed twice in ddH_2_O. Slides were then counterstained with Light Green (BioVision), washed in 100% ethanol twice for dehydration, cleared with ROTI-Histol (Carl Roth), and mounted with ROTI-Histokit (Carl Roth).

### TdT-mediated dUTP-biotin nick end labeling.

TUNEL was conducted as described in the Click-iT TUNEL Colorimetric IHC Detection kit protocol (Invitrogen [89]), counterstained with Light Green (Biovision), dehydrated in 100% EtOH, and mounted with ROTI-Histokit (Carl Roth) before microscopy. To prove correct TUNEL, a control sample treated with 200 μL DNase I (1 unit; Sigma-Aldrich), diluted in 1× DNase reaction buffer (Table S3), was included as recommended in the manufacturer’s instructions.

### Fluorescence staining.

To characterize the intercellular junctions, we performed immunofluorescence staining to visualize the respective markers, claudin-1, occludin, and ZO-1 in cryo-sections of uninfected 3D FT skin models. The staining was performed according to the protocol of Phenion FT Skin Model Histology Immunofluorescent Labeling, version 10.20 (Henkel AG [90]). The primary antibodies anti-claudin-1 (sc-166338), anti-occludin (sc-133256), and anti-ZO-1 (sc-33725; Santa Cruz Biotechnology, Dallas, TX, USA) were added in a concentration range of 1.0 to 5.0 μg · mL^−1^ after antigen blocking of the cryo-sections. The secondary antibody m-IgGκ BP-CFL 488 (sc-516176; Santa Cruz Biotechnology) was used at a concentration of 2.5 μg · mL^−1^. Cryo-sections of models exposed to LY or AFP-Bd were fixed in 4% (vol/vol) PFA and washed with PBS. All fluorescence-stained samples were mounted with Fluoroshield with DAPI (Sigma-Aldrich) for microscopic evaluation.

### Microscopy and image processing.

The fluorescence dyed samples were observed with a Zeiss Axioplan fluorescence microscope (Zeiss, Oberkochen, Germany) equipped with an Axiocam 503 mono microscope camera (Zeiss) and excitation/emission filters at 365/420 nm for DAPI, 428/536 nm for LY and CFL 488, and 546/590 nm for Bd. Histochemical staining samples were analyzed with a Zeiss Axioplan 2 microscope (Zeiss) equipped with an Axiocam 503 color microscope camera (Zeiss). All images were processed using ZEN 2 (blue edition) microscope software (Zeiss), GNU Image Manipulation Program (GIMP, version 2.8.10), and Microsoft Power Point (Microsoft Cooperation, Albuquerque, NM, USA). The LY fluorescence signal intensity in the skin sections was semi-quantified by ImageJ software (U.S. National Institutes of Health, Bethesda, MD, USA). To this end, the gray values in the *stratum corneum* and the viable epidermis were determined in at least nine sections per skin model.

### Enzyme-linked immunosorbent assay.

For the ELISA, 150 μL CCM was taken from the skin models every 24 h (ALI 7 and ALI 8 models) and stored at −80°C. The CCM was diluted 1:500 for IL-8 and 1:1,000 for IL-6 and used in a human IL-8 and IL-6 BD OptEIA ELISA (BD Biosciences, San Diego, CA, USA), as described in the manufacturer’s instructions. Readings were taken at an absorbance wavelength of 450 nm in a multi-mode plate reader (FLUOstar Omega). CCM from the uninfected/untreated models was used as a control and pure culture medium served as a blank. The amount of cytokines was calculated based on the standard curve.

### Statistical analysis.

Statistical analysis of the cytokine release, LY fluorescence signal intensity, and CFU quantification was conducted using Prism 9.1.0 (216; GraphPad Software, San Diego, CA, USA). Values are given as mean ± standard deviation (SD) of three independent skin models per experimental setting (*n* = 3). Statistical significance between the samples and controls was determined by one-way analysis of variance with a Tukey’s multiple-comparison test (*, *P* < 0.05; **, *P* < 0.005).
