# Supplementary material for: Small, Cationic Antifungal Proteins from Filamentous Fungi Inhibit Candida albicans Growth in 3D Skin Infection Models
Source: Microbiol Spectr. 2022 May 2;10(3):e00299-22. doi: 10.1128/spectrum.00299-22 (PMC9241769; doi:10.1128/spectrum.00299-22)
Supplement: SUPPLEMENTAL FILE 1 — Supplemental material. Download spectrum.00299-22-s001.pdf, PDF file, 1 MB [file spectrum.00299-22-s001.pdf]

Supplemental Material

Small, cationic antifungal proteins from filamentous fungi inhibit *Candida albicans* growth in 3D skin infection models

Jeanett Holzknecht[a], Sandrine Dubrac[b], Sarah Hedtrich[c], László Galgóczy[d,e], Florentine Marx[a#]

[a]Biocenter, Institute of Molecular Biology, Medical University of Innsbruck, Innsbruck, Austria

[b]Department of Dermatology, Venereology and Allergology, Medical University of Innsbruck, Innsbruck, Austria

[c]Faculty of Pharmaceutical Sciences, Faculty of Pharmaceutical Sciences, The University of British Columbia, Vancouver, BC, Canada

[d]Department of Biotechnology, Faculty of Science and Informatics, University of Szeged, Szeged, Hungary

[e]Institute of Biochemistry, Biological Research Centre, Eötvös Loránd Research Network, Szeged, Hungary

Running Title: Efficacy of antifungal proteins in 3D skin model

[#]Address correspondence to Florentine Marx, florentine.marx@i-med.ac.at.

Table S1. Control of the *C. albicans* inoculum for the infection of the 3D FT skin model[§].

Controls	CFU
Inoculum	200 ± 12.5
Applicator	8 ± 2

[§]A 24 h culture of *C. albicans* in YPD was washed twice in PBS and set to 1.6×10^4 cells mL^{-1} in PBS. Twenty-five µL of the cell suspension resulted in the growth of 200 CFU on SBA after incubation for 24 h at 37°C, which corresponded to the CFU also applied onto the skin models. To control the efficient application of yeast cells onto 3D FT skin models, the glass applicators were streaked over the SBA after use and the plate was incubated for 24 h at 30°C before CFU counting.

Table S2. AFPs used in this study.

Compound	Producing organism	IC_{90} [µg mL^{-1}/µM][§]	Reference
PAF^{opt}	*P. chrysogenum*	8.2/1.3	(1)
PAFB	*P. chrysogenum*	6.5/1.0	(2)
PAFC	*P. chrysogenum*	16.6/2.5	(3)
NFAP2	*N. fischeri*	2.2/0.4	(4)
Fluconazole (FLC)[$]	-	2.0/6.4	(3)

[§]Concentrations of antifungal compounds (in µg mL^{-1}/µM) that inhibit the growth of *C. albicans in vitro* by ≥90%. [$]For comparison, the IC_{90} of the antifungal drug FLC was determined under the test conditions applied in this study.

Table S3. Media and solutions used in this study.

**Media/ Solutions**	**Composition[1]/Company[2]**
DNase reaction buffer	20 mM Tris-HCl, pH 8.4, 2 mM $MgCl_2$, 50 mM KCl
GMS working solution	1.5% methenamine, 0.125% silver nitrate, 0.2% Borax
Lysogeny broth (LB) agar	1% NaCl, 1% neutralized bacterial peptone, 0.5% yeast extract, 2% agar
Phosphate buffered saline (PBS)	0.5% KH_2PO_4, 2.8% K_2HPO_4, 9% NaCl
Potato dextrose broth (PDB)	
Potato dextrose agar (PDA)	PDB, 2% agar
Sabouraud agar (SBA)	1% peptone, 4% D-(+)-glucose, 2 % agar, pH 5.6
Yeast extract peptone dextrose (YPD) medium	1% yeast extract, 2% bacteriological peptone, 2% D-(+)-glucose
Yeast extract peptone dextrose (YPD) agar	YPD, 2% agar

[1]Values (%) are depicted as (wt/vol) unless otherwise stated. [2]All chemicals and compounds were purchased from Sigma-Aldrich, St. Louis, MO, USA.

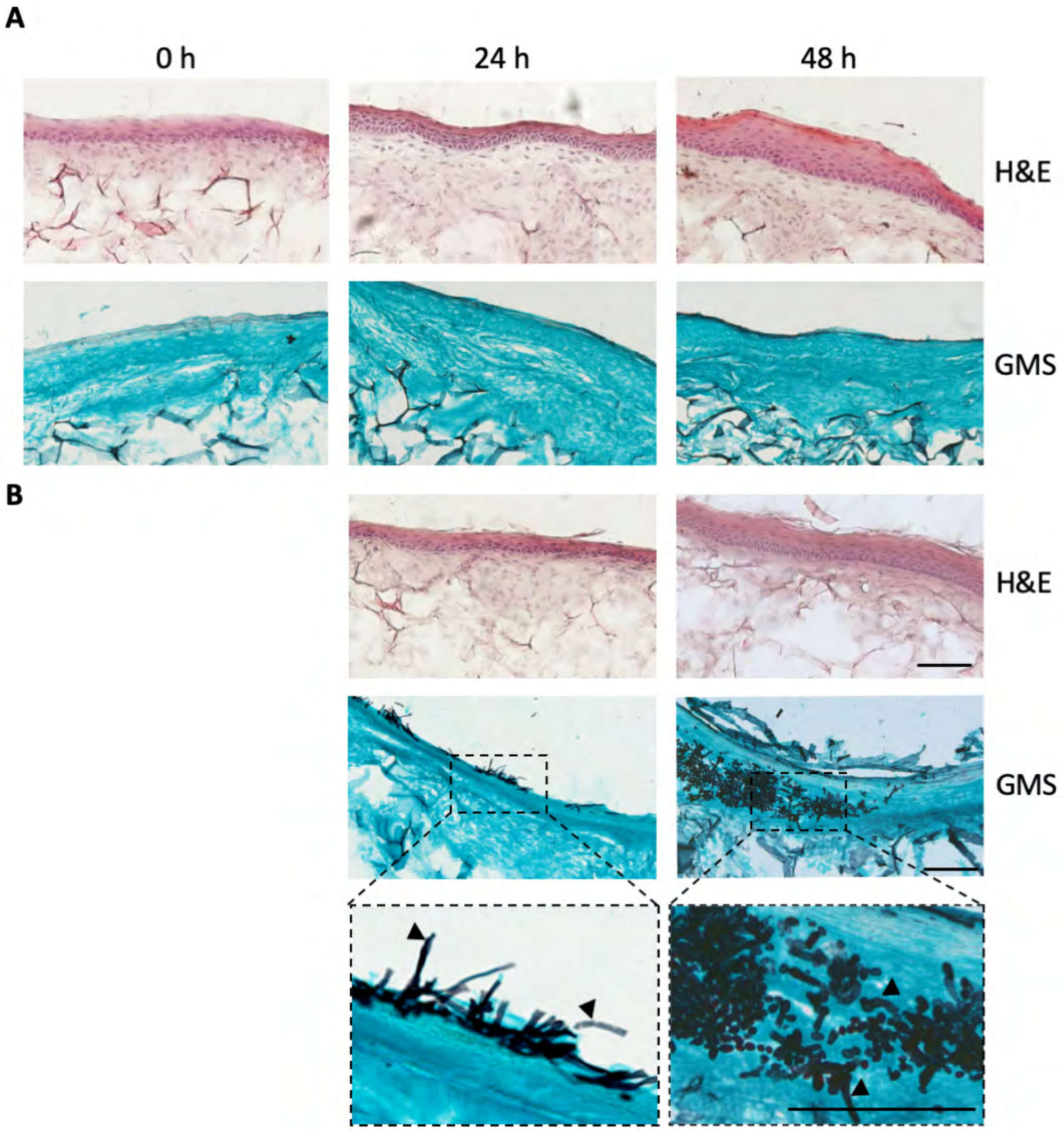

Figure S1. ***C. albicans* infection in a 3D FT skin model.** Skin models **(A)** without and **(B)** with *C. albicans* infection were analyzed after 0 h, 24 h and 48 h of incubation at 32°C, 5% CO_2. Cryo-sections were stained with H&E and GMS before microscopy. Colonization of the tissue with *C. albicans* is marked with arrowheads. Scale bars, 100 µm.

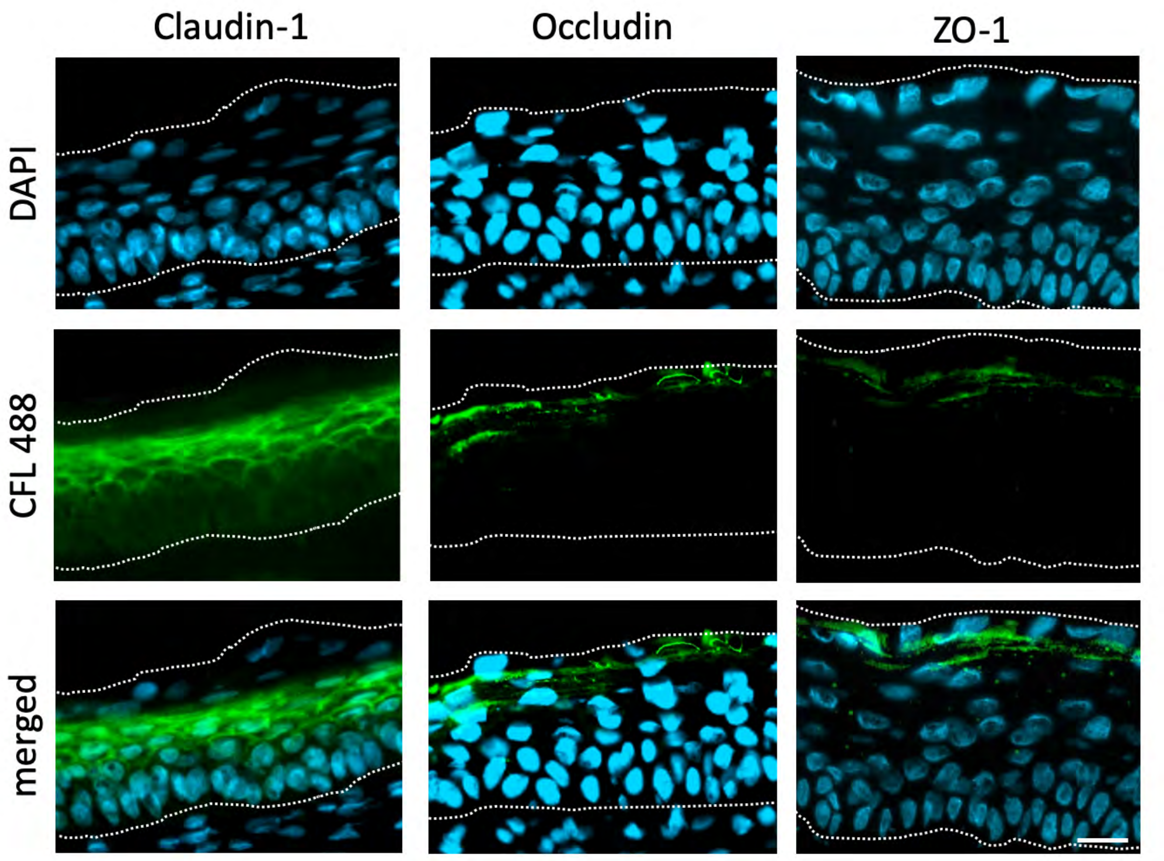

Figure S2. Display of intercellular junctions in the epidermal layer of the Phenion 3D FT skin models. Immunofluorescence staining of skin models show tight junction proteins claudin-1, occludin and ZO-1 (green, respectively). Cryo-sections were mounted with Fluoroshield™ with DAPI before microscopy. Dotted lines delineate the epidermis of the models. Scale bar, 100 µm.

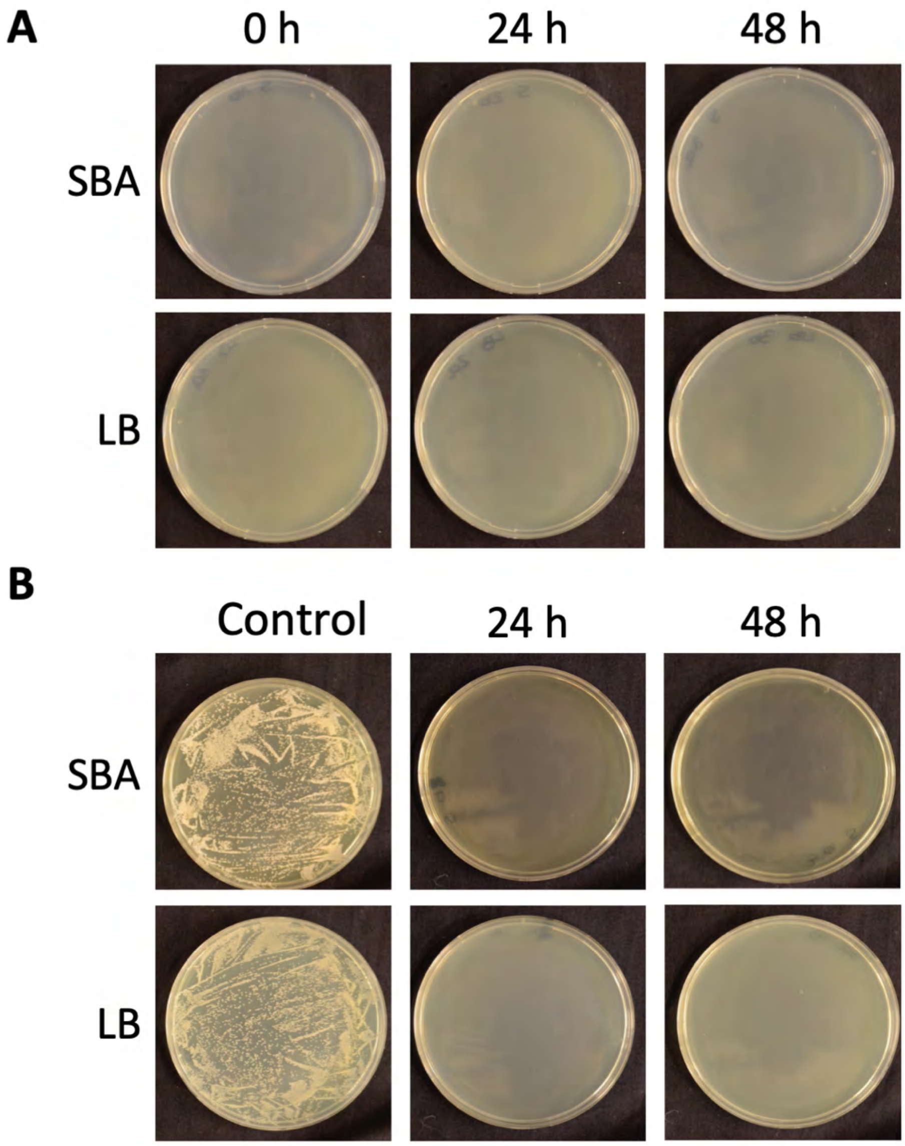

Figure S3. Analysis of potential microbial contamination of the CCM after cultivation of the 3D FT skin model. CCM was collected from ALI 6 (0 h), ALI 7 (24 h) and ALI 8 (48 h) skin models **(A)** without and **(B)** with *C. albicans* infection and 50-µL aliquots were plated on SBA or LB agar to detect possible bacterial or fungal contamination. *C. albicans* was plated as a growth control. The agar plates were incubated at 37°C for 24 h and documented with a Nikon digital camera D7000.

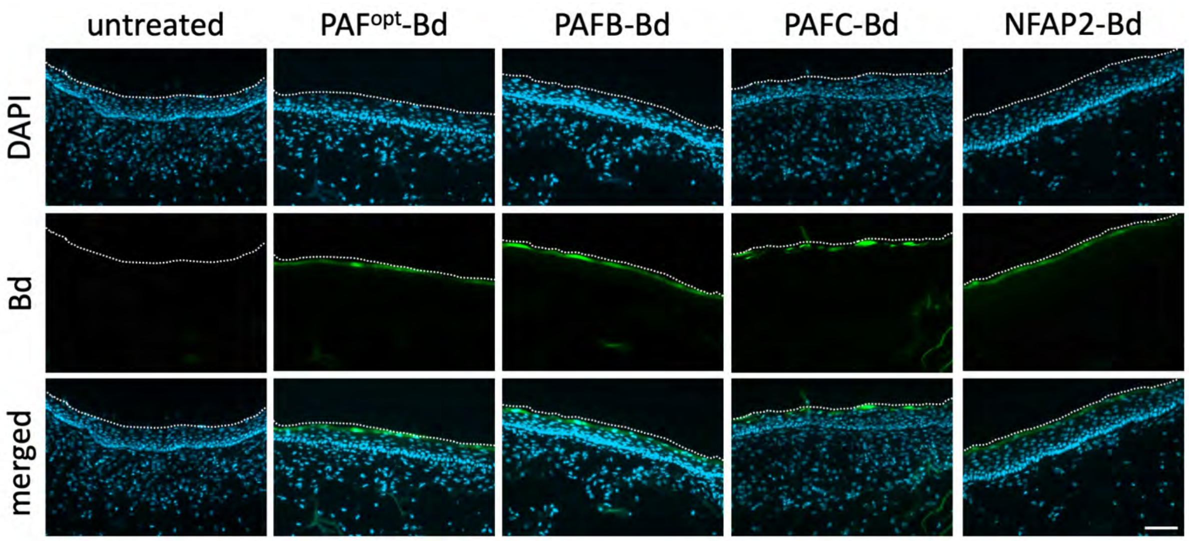

Figure S4. Localization of AFPs in the 3D FT skin model. Bd-labelled AFPs (PAF^{opt}-Bd, PAFB-Bd, PAFC-Bd (18 mg mL^{-1} each); NFAP2-Bd (6.4 mg mL^{-1})) were topically applied in 25 µL aliquots on 3D FT skin models and incubated for 24 h at 32°C, 5% CO_2 in the dark. The control model was treated with 25 µL ddH_2O (untreated). Cryo-sections were mounted with Fluoroshield™ with DAPI before microscopy. Dotted lines delineate the skin surface of the models. Scale bar, 100 µm.

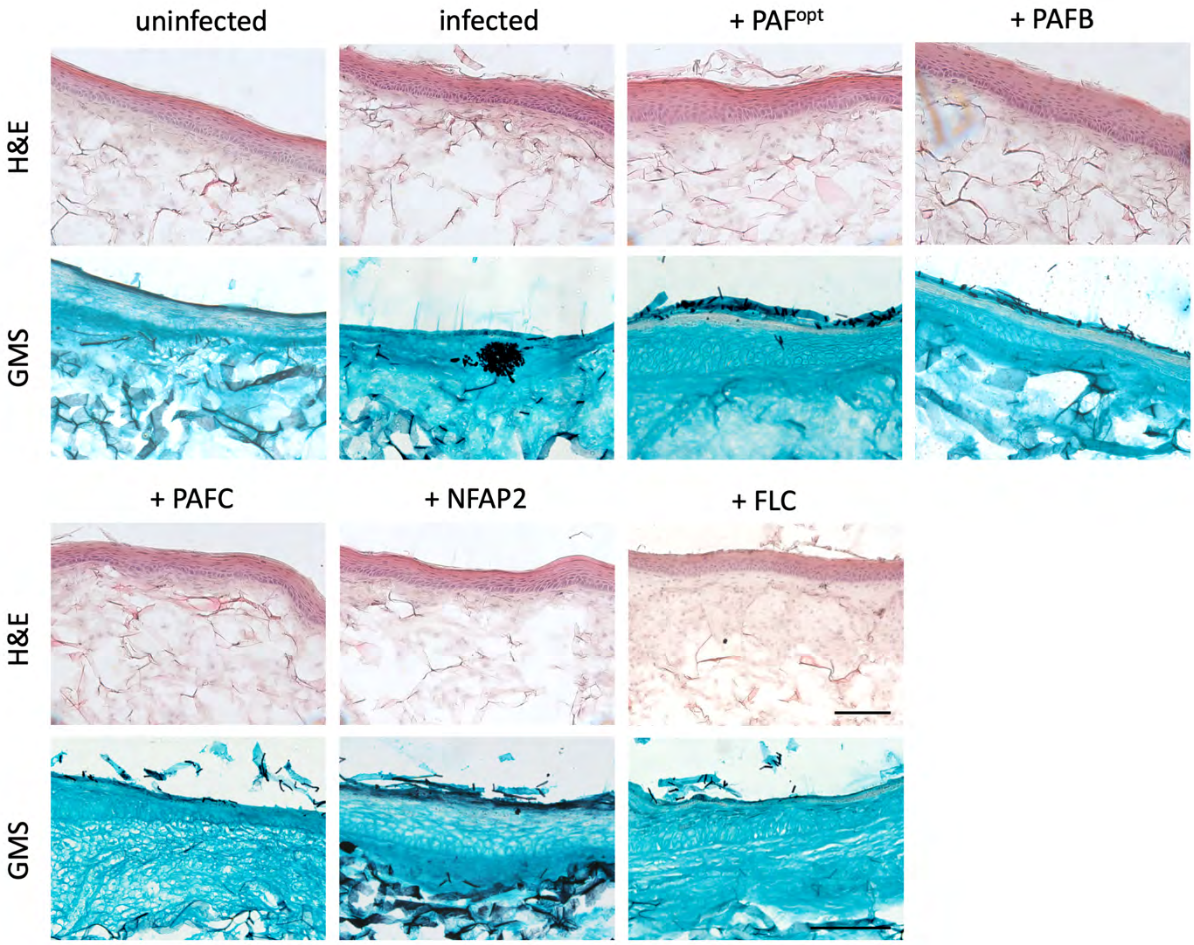

Figure S5. ***C. albicans* infection and treatment with AFPs in a 3D FT skin model. (A)** H&E and GMS stained cryo-sections of skin models that were infected with *C. albicans* and incubated for 24 h at 32°C, 5% CO_2. Then 25 µl AFPs (PAF^{opt}, PAFB, PAFC (18 mg mL^{-1} each)); NFAP2 (6.4 mg mL^{-1}) and FLC (0.02 mg mL^{-1}), respectively, were topically applied before the models were further incubated for 24 h under the same cultivation conditions. Untreated, uninfected (untreated) and infected models without treatment (*C. albicans*) served as controls. Scale bars, 100 µm.

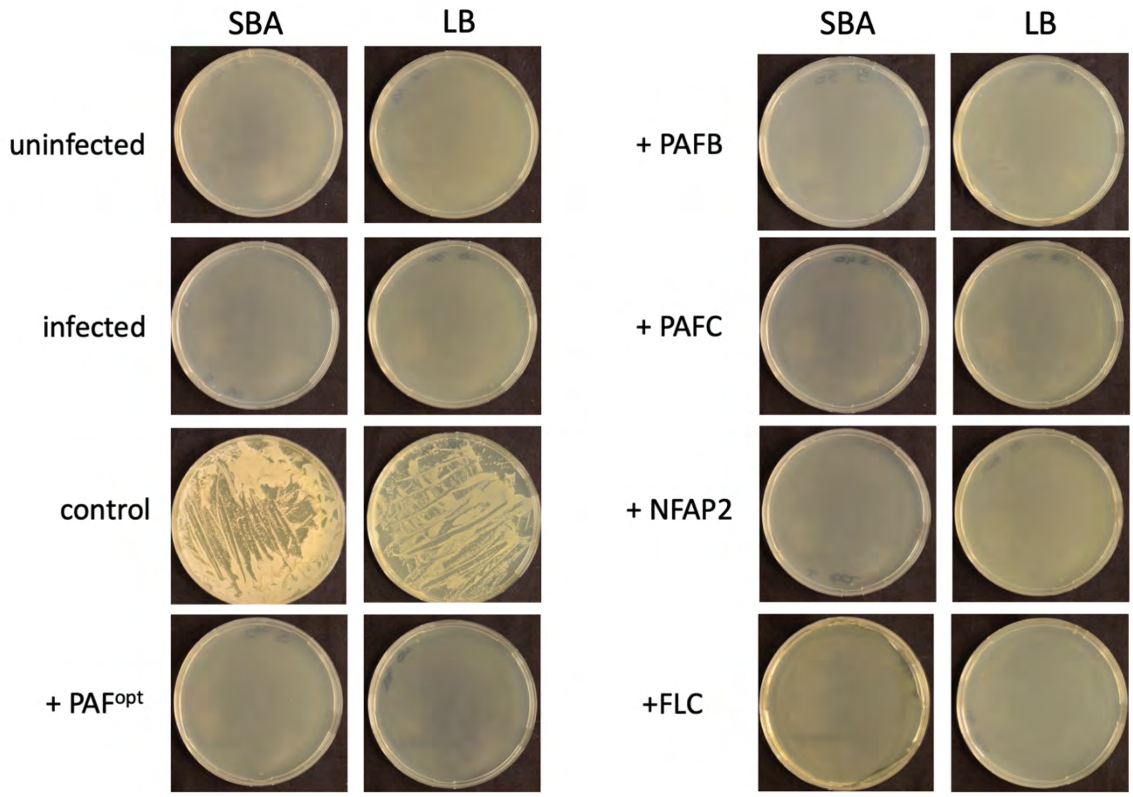

Figure S6. Analysis of potential microbial contamination of the CCM after cultivation of the 3D FT skin model. The CCM was collected from the 3D FT skin models after the distinct treatments and 50 µL aliquots were plated on SBA or LB agar to detect possible bacterial or fungal contamination. For growth control, *C. albicans* cells were plated. The plates were incubated at 37°C for 24 h and documented with a Nikon digital camera D7000.

Supplemental References

1. Sonderegger, C., Váradi, G., Galgóczy, L., Kocsubé, S., Posch, W., Borics, A., Dubrac, S., Tóth, G. K., Wilflingseder, D., Marx, F. (2018). The evolutionary conserved γ-core motif influences the anti-*Candida* activity of the *Penicillium chrysogenum* antifungal protein PAF. *Front Microbiol*, 9, 1655. doi: 10.3389/fmicb.2018.01655
2. Huber, A., Hajdu, D., Bratschun-Khan, D., Gáspári, Z., Varbanov, M., Philippot, S., Fizil, Á., Czajlik, A., Kele, Z., Sonderegger, C., Galgóczy, L., Bodor, A., Marx, F., Batta, G. (2018). New antimicrobial potential and structural properties of PAFB: a cationic, cysteine-rich protein from *Penicillium chrysogenum* Q176. *Sci Rep*, 8(1), 1751. doi: 10.1038/s41598-018-20002-2
3. Holzknecht, J., Kühbacher, A., Papp, C., Farkas, A., Váradi, G., Marcos, J. F., Manzanares, P., Tóth, G. K., Galgóczy, L., Marx, F. (2020). The *Penicillium chrysogenum* Q176 antimicrobial protein PAFC effectively inhibits the growth of the opportunistic human pathogen *Candida albicans*. *J Fungi (Basel)*, 6(3), 141. doi: 10.3390/jof6030141
4. Tóth, L., Kele, Z., Borics, A., Nagy, L. G., Váradi, G., Virágh, M., Takó, M., Vágvölgyi, C., Galgóczy, L. (2016). NFAP2, a novel cysteine-rich anti-yeast protein from *Neosartorya fischeri* NRRL 181: isolation and characterization. *AMB Express*, 6(1), 75. doi: 10.1186/s13568-016-0250-8
